# Bimetallic PdAu Catalysts within Hierarchically Porous Architectures for Aerobic Oxidation of Benzyl Alcohol

**DOI:** 10.3390/nano11020350

**Published:** 2021-02-01

**Authors:** Priyanka Verma, Matthew E. Potter, Alice E. Oakley, Panashe M. Mhembere, Robert Raja

**Affiliations:** School of Chemistry, University of Southampton, University Road, Highfield, Southampton SO17 1BJ, UK; P.Verma@soton.ac.uk (P.V.); M.E.Potter@soton.ac.uk (M.E.P.); A.E.Oakley@soton.ac.uk (A.E.O.); P.M.Mhembere@soton.ac.uk (P.M.M.)

**Keywords:** noble metal nanoparticles, alcohol oxidation, bimetallic nanoparticles, hierarchically porous materials

## Abstract

Hierarchically porous (HP) zeotype materials (possessing both micropores and mesopores) offer improved diffusional access to intra-framework active sites, analogous to mesoporous materials, yet retain the high selectivity of the microporous (MP) bulk. We have recently designed crystalline hierarchically porous silicoaluminophosphates (SAPOs) with enhanced mass-transport characteristics, which can lead to significant improvement in catalytic activity and catalyst lifetime. In this study, we have prepared PdAu bimetallic nanostructures supported on HP-SAPO frameworks by an incipient impregnation of metal precursors followed by H_2_ reduction at 300 °C, for the aerobic oxidation of benzyl alcohol to benzaldehyde. PdAu NPs supported on HP framework displayed significantly enhanced catalytic activities, when compared with their MP analogues, clearly highlighting the benefits of introducing hierarchical porosity in the SAPO support matrix.

## 1. Introduction

The pioneering research work by Haruta et al. led to the discovery of Au NPs as an active catalyst in oxidation reactions at relatively low temperatures [1,2]. Since then, they have been extensively studied in various organic transformation reactions such as controlled oxidation of alcohol, direct synthesis of H_2_O_2_ and selective hydrogenation reactions [3,4,5,6,7,8,9,10]. Many research reports have investigated that the textural and chemical properties of support materials, type of metal and size of NPs directly influence the catalytic activity and selectivity of oxidation reaction (Table S1) [11,12,13,14,15,16]. The Au NPs supported on metal oxides (TiO_2_, CeO_2_, Al_2_O_3_, MgO) and carbon materials [17,18,19] have been reported to be the active catalysts for alcohol oxidation reactions [7,20,21,22]. Many research groups have explored the use of Au [23], Pd [24,25] and PdAu [26] bimetallic NPs on these support materials [20,21,22,23,27,28]. The oxide supported bimetallic catalysts, in particular the combination of Pd and Au, has been recognised for its highly active and selective oxidation abilities in various important catalytic transformations by weakly adsorbing the reactants and products on its surface to form target molecules [24,25,26,29,30,31]. These bimetallic catalysts are advantageous not only because of their electronic interactions but also due to the possible complex geometrical arrangements, charge transfer and interfacial stabilisation between Pd and Au atoms. 

Unlike traditional metal oxides, the use of mesoporous and microporous support materials offers the confined growth of metal NPs by giving stability and high dispersion especially for Au NPs, which tends to accumulate to form larger aggregates [32,33,34,35,36]. The immobilisation of metal NPs on microporous supports by impregnation followed by H_2_ reduction at high temperature, for example, zeolites and aluminophosphates (AlPOs), can assist in achieving targeted product selectivity. However, their small pore size can restrict the access of substrate molecules to the active sites and hence leads to lower conversion yields. On the contrary, mesoporous materials can retain their porosity after NPs incorporation but lack the more subtle ability to control the space around active site. This shortcoming led to the synthesis of novel hierarchically porous (HP) materials, which combine the advantages of targeted selectivity in microporous systems along with improved diffusion ability in mesoporous materials. Ryoo et al. pioneered the synthesis of hierarchical AlPOs by hydrothermal assembly process in which organosilanes surfactants self-assemble to form micelles along with the generation of the microporous framework [37]. The micelles and surface-directing agents were removed by calcination to create HP-AlPO framework with silanol lined mesopores. In our previous reports, we have successfully demonstrated the synthesis of HP framework by adding dimethyloctadecyl[(3-(trimethoxysilyl)propyl] ammonium chloride (DMOD) micellar agent, along with silica source during the AlPO synthesis, in which silanol lined mesopores were formed along with micropores to generate HP silicoaluminophosphate (SAPO) frameworks [38,39,40,41]. The amount of Si loading was fixed in order to avoid Si islanding within the framework, which lowers the overall number of active sites [42]. The traditional microporous (MP) SAPO-5 framework can be synthesised following the same procedure in the absence of DMOD [43]. 

In this study, we seek to specifically probe the anchoring of monometallic and bimetallic nanocatalysts on analogous microporous and hierarchical SAPO-5 systems, to rationalise the overall stability of these supports. The microporous SAPO-5 catalysts possess Al-OH, P-OH and Si-OH sites on the exterior of the micropores, and based on previous studies [38,41,44,45], these provide sites for anchoring of nanoparticles, based on weak, van-der-Waals interactions. On the other hand, the soft-templating synthetic strategy that have evolved for the HP-SAPO-5 analogues [38], affords pendant Si-OH groups, that line the internal walls of the mesopores, which are proficient for the covalent anchoring of nanoparticles, akin to mesoporous silica supports [46,47,48,49,50,51,52,53]. Through this approach, we can probe the efficacy of the anchoring of nanoparticles to these internal and external binding sites in MP-SAPO-5 and HP-SAPO-5 catalysts, thereby highlighting their relative merits in the heterogenization of metal nanoparticles. Whilst our previous report highlights the preservation of porosity within hierarchically porous AlPOs after NP incorporation [40], this study specifically probes the relative stability of the microporous and hierarchical SAPO-5, with respect to nature of anchoring of metal nanoparticles. The synthesis and stabilisation of PdAu bimetallic nanostructures within hierarchically porous silicoaluminophosphates (HP-SAPO-5) has also not been reported in the literature so far. The efficacy of prepared catalysts was tested in the aerobic oxidation of benzyl alcohol to benzaldehyde at 100 °C and atmospheric pressure. The choice of this model reaction serves to vindicate the overall objectives of this study, as outlined above, and paves the way for the exploitation of the anchoring methodology within these novel hierarchical systems, which can aid the future exploitation of these catalysts for more demanding catalytic reactions (selective oxidations and hydrogenations). Furthermore, with our adroit choice of monometallic and bimetallic catalysts, we are also able to demonstrate the superior properties of the bimetallic HP-SAPO-5, when compared with its monometallic analogue and comparative physical mixture variant. 

## 2. Materials and Methods 

### 2.1. Materials

Phosphoric acid (H_3_PO_4_, 85% in H_2_O), Aluminium isopropoxide (Al[OCH(CH_3_)_2_]_3_), Dimethyloctadecyl[(3-(trimethoxysilyl)propyl] ammonium chloride, DMOD, triethylamine (N(CH_2_CH_3_)_3_), tetrachloroauric (III) acid (HAuCl_4_), palladium (II) acetate (Pd(OAc)_2_), Ethanol (C_2_H_5_OH), Benzyl alcohol (C_7_H_8_O), Benzaldehyde (C_7_H_6_O) and Biphenyl (C_6_H_5_)_2_ were bought from Merck chemicals (Darmstadt, Germany). All chemicals were used as such without any further purification. 

### 2.2. Synthesis of Hierarchically Porous SAPO-5 Support Materials

HP-SAPO-5 and MP-SAPO-5 were synthesised as per our previously reported literature [38]. Typically, for HP-SAPO-5, phosphoric acid (2.28 mL, 85% in H_2_O), and water (10 mL) mixture was prepared and stirred continuously. To this acidic mixture, finely grounded aluminium isopropoxide (6.807g) was slowly added until homogeneous. Subsequently, the dropwise addition of dimethyloctadecyl[(3-(trimethoxysilyl)propyl] ammonium chloride, DMOD, (1.2 mL, 72% in H_2_O), triethylamine (3.7 mL, Aldrich) and water (20 mL) was carried out and stirred vigorously for 1 h. The Si source, silica sol (0.771 mL, 40% in water) was then added, and the mixture was stirred for 1.5 h to obtain a gel with the following composition:

1.0 Al: 1.0 P: 0.8 TEA: 50 H_2_O: 0.15 Si: 0.05 DMOD

The white gel was then transferred into the stainless-steel autoclave and treated hydrothermally for 24 h at 200 °C. The obtained white solid was collected by filtration and washed with 500 mL of deionized water. After drying the product overnight at 80 °C, it was calcined in a tube furnace under a continuous flow of air at 550 °C for 16 h. The white crystalline solid was labelled as HP-SAPO-5. The MP-SAPO-5 was also synthesised by using the equivalent method in the absence of DMOD during the synthesis procedure.

### 2.3. Synthesis of PdAu Bimetallic Nanocatalyst within HP-SAPO-5 Architectures

PdAu bimetallic nanocatalyst confined within HP-SAPO-5 support was synthesised via two simple steps of impregnation followed by H_2_ reduction at 200 °C. At first, 0.2 g of support material (HP-SAPO-5) was dispersed in ethanol and ultrasonicated until homogeneous. The required amount of HAuCl_4_ aqueous solution was added and stirred continuously for 4 h. The solvent was then evaporated using rotavapor and the obtained powder was dried overnight at 80 °C. The yellowish impregnated powder was then reduced under 5% H_2_/N_2_ mixture in a tube furnace for 4 h at 300 °C to obtain Au/HP-SAPO-5 with a target metal content of 1.0 wt%. Subsequently, Pd was deposited by adding Pd(OAc)_2_ in the ethanolic suspension of Au/HP-SAPO-5 and reduced in the same way as for Au NPs to form PdAu/HP-SAPO-5. The monometallic Pd/HP-SAPO-5 was also prepared for the comparison of catalytic activities.

### 2.4. Catalytic Reaction

The aerobic oxidation reaction of benzyl alcohol to benzaldehyde under the flow of air (50 mL min^−1^) was carried out as a model reaction to study the activity of the prepared PdAu catalysts. Twenty milligrams of PdAu/HP-SAPO-5 catalyst was weighed and added into the glass reactor along with 1 mmol of benzyl alcohol and 0.5 mmol of K_2_CO_3_ to ensure the basic medium of the reaction. 0.25 mmol of biphenyl (external standard reagent) was added for calibrating the concentration of reactants and products during the course of the reaction. Twenty-five millilitres of toluene were added, and the reactor was magnetically stirred at 100 °C in an oil bath. The reaction was initiated by controlling the airflow using a mass-flow controller at 50 mL min^−1^ through the reaction mixture. The time course of reaction products was analysed by using Clarus 480 gas chromatograph equipped with a flame ionisation detector and Elite 5 column. The reaction products were monitored every hour for 6 h and products were identified and quantified against external standard (biphenyl) reagent. 

### 2.5. Characterisation

Perkin Elmer Lambda 35 spectrophotometer (Waltham, MA, USA) was used to collect the reflectance UV–vis spectra of powdered samples. BaSO_4_ was used as a reference solid material. Brunauer–Emmett–Teller (BET) surface area measurement was performed by using a Micromeritics Gemini Tristar 3020 (Norcross, GA, USA) surface area analyser at −196 °C. Degassing of the samples was done in a vacuum at 150 °C for 6 h in order to remove the adsorbed impurities. TEM micrographs were obtained with the FEI Tecnai T12 (Hillsboro, OR, USA) instrument at Biomedical Imaging Unit, University Hospital Southampton. Clarus 480 gas chromatograph equipped with a flame ionisation detector and Elite 5 column was used to analyse reaction products employing biphenyl as an external standard reagent. Powder X-ray diffraction (XRD) patterns were collected by using a Bruker D2 diffractometer (Waltham, MA, USA) using Cu K α1 radiation. The metal content was determined by ICP analysis using high-resolution inductively coupled plasma-mass spectrometer (ICP-MS) Thermo Scientific ELEMENT 2XR (Waltham, MA, USA), with appropriate standards for quantification. Pd K-edge and Au L_3_-edge XAS studies were carried out on the B18 beamline (session ID SP19850-5) at the Diamond Light Source as part of our membership of the UK Catalysis Hub Beamtime Allocation Group. XPS was measured at Harwell XPS, an EPSRC national facility for X-ray photoelectron spectroscopy. The obtained spectra were calibrated against C1s peak at 285 eV.

## 3. Results and Discussion

### 3.1. Structural Characterisation

**Figure 1:**
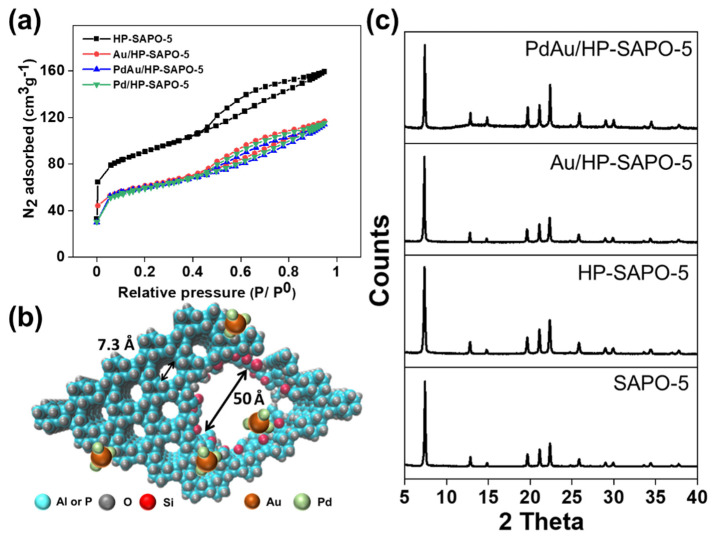
(**a**) N_2_ physisorption analysis and (**b**) Illustration of PdAu/HP-silicoaluminophosphates (SAPO)-5 (**c**) X-ray diffraction pattern of prepared catalysts.

The HP-SAPO-5 support material was synthesised by a one-pot soft templating strategy in which a mesporogen; DMOD, was used in combination with a microporous structure-directing agent, triethylamine (TEA) [38]. The Al, P and Si atoms were then self-assembled to generate a HP architecture after calcination. The generated silanol sites line the mesopores of the SAPO framework, which assists in stabilising the formation of metal NPs. The microporous support was also synthesised following a similar procedure in the absence of DMOD reagent to form MP-SAPO-5. The prepared catalysts were characterised by a range of physicochemical characterisation techniques including powder-XRD, N_2_ physisorption, Fourier Transform-extended X-ray absorption fine structure (FT-EXAFS), TEM and UV-vis spectroscopy. The synthesis and design strategy of HP-SAPO-5 and MP-SAPO-5 support materials was confirmed by N_2_ physisorption analysis. As shown in Figure 1a, HP-SAPO-5 displayed type IV isotherm indicating the presence of microporous framework associated with the mesoporous network. The MP-SAPO-5 exhibited type I isotherm corresponding to microporous architectures as shown in Figure S1. The BET surface areas of MP-SAPO-5 and HP-SAPO-5 were found to be 271 m^2^g^−1^ and 299 m^2^g^−1^ respectively, in agreement with reported literature [38].

The calcined HP-SAPO-5 was then used as a support for depositing Au and Pd nanoparticles, both separately (Au/HP-SAPO-5 and Pd/HP-SAPO-5) and on the same support (PdAu/HP-SAPO-5), to create a unique bimetallic system as illustrated in Figure 1b. In all cases, this led to a decrease in total surface area (from 299 m^2^g^−1^ to 199-194 m^2^g^−1^, Table 1), and a slight reduction in pore volume (Figure S1), though the average mesopore diameter was retained (Figure S2). We note that both the micropore and mesopore pore volume drop by roughly one third, suggesting that both are influenced by the binding process. The HP-SAPO-5 framework anchored with monometallic (Pd, Au) and bimetallic (PdAu) catalysts displayed similar porosity characteristics (Table 1). This suggests that any differences in the catalytic performances between the doped HP-SAPO-5 species will not be a function of porosity and reactant accessibility. For comparison, the microporous SAPO-5 species was also impregnated with both Au and Pd nanoparticles to create an analogous species; PdAu/MP-SAPO-5. Unlike the hierarchical species, the microporous MP-SAPO-5 support lost a significant amount of porosity on depositing Pd and Au simultaneously (PdAu/MP-SAPO-5, Table 1). Specifically, we note a large decrease in surface area (from 271 to 36 m^2^g^−1^) and also in micropore pore volume (from 0.12 to 0.02 cm^3^g^−1^). The retention of porosity in HP-SAPO-5 systems, on metal deposition, shows the advantages of introducing mesoporosity to the system. Further, the silanols introduced by the DMOD can provide an alternative binding site, directing some of the NPs away from the micropores, or pore openings, preventing micropore blockage [47,48,49,51,52]. The structural integrity after NPs incorporation was investigated by the powder XRD pattern of all samples. It was observed that monometallic (Au/HP-SAPO-5) and bimetallic (PdAu/HP-SAPO-5) nanoarchitectures preserved their crystallinity and phase purity as shown in Figure 1c. 

**Figure 2:**
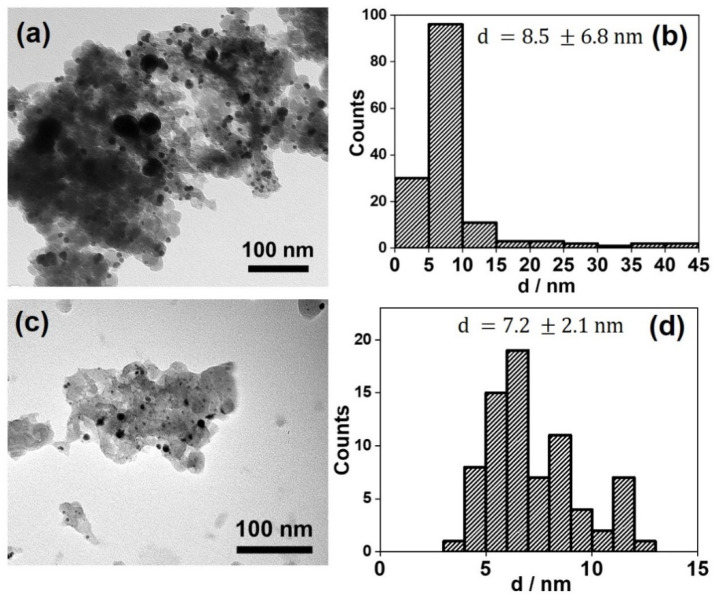
TEM micrographs and size distribution histograms of (**a**,**b**) PdAu/HP-SAPO-5 and (**c**,**d**) PdAu/MP-SAPO-5.

The NP size distributions were determined by analysing transmission electron microscope (TEM) images of calcined catalysts. Figure 2a–d displays the TEM micrographs and associated histograms of PdAu/HP-SAPO-5 and PdAu/MP-SAPO-5. The two systems showed similar average nanoparticle diameters, at 8.5 ± 6.8 and 7.2 ± 2.1 nm for PdAu/HP-SAPO-5 and PdAu/MP-SAPO-5, respectively, both presenting spherical and isolated NPs. It was observed that NPs were highly dispersed on the surface of hierarchically porous architectures in comparison to the microporous supports as shown in Figure 2. Elemental mapping of PdAu/HP-SAPO-5 was used to explore the distribution of Al, P, Si, Au and Pd (Figure S3). Al, P and Si were prevalent throughout as primary components of the SAPO-5 supports (Table 2). The compositions of Au and Pd within the SAPO framework also quantitatively match with the synthesis ratio within 1 weight%. The PdAu representative line profiles in the energy dispersive X-ray (EDX) spectra also depicted intense peaks, further confirming the atomic presence of Al, O, P, Si, Au and Pd within the energy range of 0–10 eV as shown in Figure S4. 

ICP analysis revealed the actual weight content of various elements incorporated in the preparation of support materials and metal NPs. The Au content varied from 0.6–0.7 wt% and Pd showed variations from 0.1–0.4 wt%. The composition of Al and P in the framework was found to be in a similar range for all prepared samples as shown in Table 2. The loading of Si varied from 3.6–9.6 wt% for MP and HP-SAPO-5.

The light absorption ability of Au and PdAu/HP-SAPO-5 catalysts was probed by using UV-vis absorption spectroscopy as summarised in Figure S5. Both PdAu/HP-SAPO-5 and PdAu/MP-SAPO-5 show a broad peak from 400–520 nm, assigned as a localised surface plasmon resonance band due to the presence of Au NPs [32]. The broad nature of this absorption could suggest the presence of a variety of deposition sites and environments within these materials. The observed reduction in intensity of the localised surface plasmon band, in the bimetallic PdAu systems, may indicate the surface covering of Au by Pd NPs [34]. 

**Figure 3:**
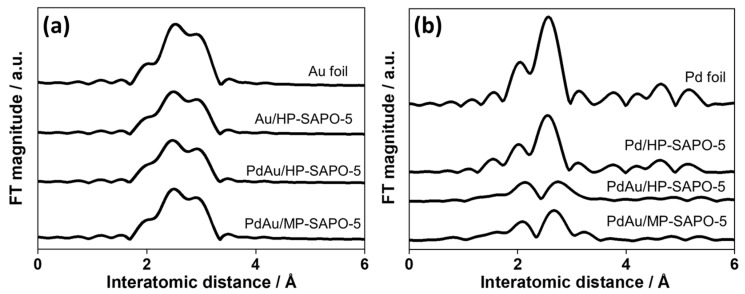
Fourier transforms of (**a**) Au L_III_-edge EXAFS spectra for Au foil (reference), Au/HP-SAPO-5, PdAu/HP-SAPO-5, PdAu/MP-SAPO-5 and (**b**) Pd K-edge EXAFS spectra for Pd foil (reference), Pd/HP-SAPO-5, PdAu/HP-SAPO-5 and PdAu/MP-SAPO-5.

X-ray absorption spectroscopy (XAS) measurement studies were carried out to probe the local structure and chemical environment of Au and Pd NPs immobilised on hierarchically porous and microporous support materials as shown in Figure 3. The Au L_III_ edge-Fourier transform extended X-ray absorption fine structure spectra (FT-EXAFS) of Au/HP-SAPO-5, PdAu/HP-SAPO-5, PdAu/MP-SAPO-5 along with Au foil as the reference sample is shown in Figure 3a. An intense peak at 2.52 Å for Au foil was also observed in all PdAu and Au catalysts. This can be ascribed to the contiguous Au-Au bonding, confirming the presence of Au^0^ species. The Au coordination number was found to be in the range of 10-11 which is lower than the theoretical value of 12 for bulk systems, indicating the formation of non-bulk Au systems. The X-ray absorption near edge structure (XANES) spectra for all catalysts is shown in Figure S6a. Au foil, Au/HP-SAPO-5 and PdAu/HP-SAPO-5 display a very similar shape of spectra and hence similar electronic properties. Figure 3b displays the Pd-K edge FT-EXAFS spectra of prepared catalysts in addition to the Pd foil as the reference material. The main peak at 2.57 Å was ascribed to the adjoining Pd-Pd metallic bond confirming the zero-oxidation state of Pd NPs. Moreover, the edge position and shape of Pd catalysts were also similar to Pd foil in XANES spectra as shown in Figure S6b.

**Figure 4:**
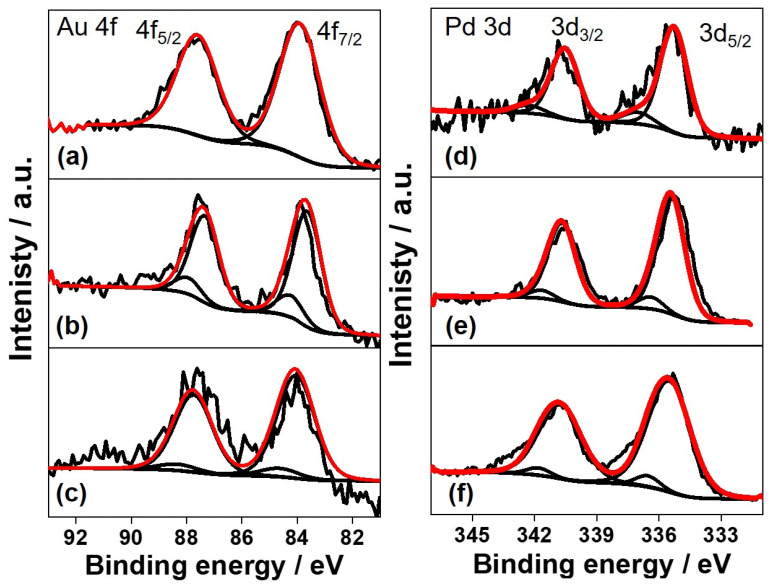
X-ray photoelectron spectroscopy (XPS) spectra of Au 4f for (**a**) Au/HP-SAPO-5, (**b**)PdAu/HP-SAPO-5, (**c**) PdAu/MP-SAPO-5 and Pd 3d spectra for (**d**) Pd/HP-SAPO-5, (**e**) PdAu/HP-SAPO-5 and (**f**) PdAu/MP-SAPO-5.

The surface composition and chemical state of Pd and Au metal NPs was studied by X-ray photoelectron spectroscopy (XPS) as shown in Figure 4. The obtained spectra were calibrated against C 1s peak centered at binding energy value of 285 eV. The Au 4f core electrons spectra for Au/HP-SAPO-5, PdAu/HP-SAPO-5 and PdAu/MP-SAPO-5 is shown in Figure 4a–c. Figure 4d–f displays the Pd 3d XPS spectra for Pd/HP-SAPO-5, PdAu/HP-SAPO-5 and PdAu/MP-SAPO-5. The occurrence of doublet peaks 4f_5/2_, 4f_7/2_ for Au/HP-SAPO-5 at 84.0, 87.6 eV and 3d_3/2_, 3d_5/2_ for Pd/HP-SAPO-5 at 335.2, 340.4 eV confirmed the metallic state of formed NPs [54,55]. The presence of oxide peaks was also observed in Pd and PdAu-based samples due to the susceptible air oxidation of metal NPs. The bimetallic Pd 3d and Au 4f core electrons displayed shift in the binding energy values towards higher and lower values, respectively in comparison to the monometallic counterparts (Table S2). The integration of Au and Pd NPs induces the charge transfer from Pd to Au because of the difference in their electronegativity values (Au: 2.54 and Pd: 2.20), making Pd more electron deficient. Hence, XPS provides a strong tool to evidence and study the surface and chemical compositions of metal NPs.

### 3.2. Catalysis of Benzyl Alcohol Oxidation

**Scheme 1:**
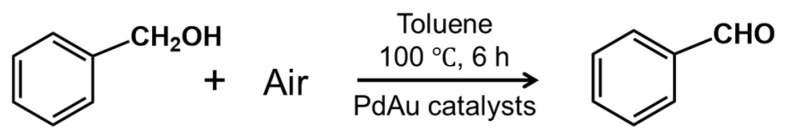
Controlled oxidation of benzyl alcohol over PdAu catalysts supported on hierarchically porous architectures.

The effect of dual-porosity on the aerobic oxidation of benzyl alcohol to benzaldehyde over PdAu bimetallic catalyst was studied by comparing the behaviour of PdAu/HP-SAPO-5 and PdAu/MP-SAPO-5 systems. The oxidation reaction over supported transition metal NPs can proceed under mild reaction conditions and accelerated by the addition of weak base [4]. The catalysis was carried out at 100 °C for 6 h in the presence of K_2_CO_3_ (base) under the continuous flow of air (Scheme 1). The quantitative yield of benzaldehyde was determined based on the peak area values of benzyl alcohol, benzaldehyde and external standard reagent biphenyl. No product formation was observed in the absence of catalyst or airflow. 

**Figure 5:**
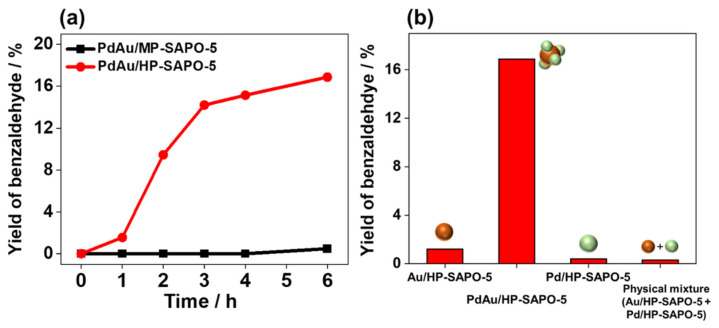
(**a**) Reaction kinetics for PdAu bimetallic catalysts supported on hierarchically porous and microporous SAPO-5 supports and (**b**) contrasting the monometallic and bimetallic nanostructures for their catalytic activity in the oxidation reaction. Reaction conditions: Catalyst (20 mg), toluene (solvent; 25 mL), benzyl alcohol (substrate; 5 mmol), K_2_CO_3_ (base; 1 mmol), biphenyl (external standard; 1.2 mmol), time (6 h) and airflow (50 mLmin^−1^).

The influence of dual porosity was investigated by comparing the bimetallic PdAu/HP-SAPO-5 and PdAu/MP-SAPO-5 systems. Figure 5a displays the reaction time profile for the benzyl alcohol oxidation over PdAu/HP-SAPO-5 and PdAu/MP-SAPO-5 catalysts. The porosity had a significant impact on the obtained catalytic performances, as the PdAu/HP-SAPO-5 shows vastly superior catalytic activity compared to the PdAu/MP-SAPO-5 system. This is primarily due to the hierarchical system (PdAu/HP-SAPO-5) retaining most of its porosity on deposition, in contrast to the microporous species (PdAu/MP-SAPO-5) as shown in Figure S1 and Table 1. We believe this is a direct consequence of the silanol-lined mesopores in the hierarchical system. These species offer alternative binding sites for the NPs, in the mesopores, preventing near-complete blockage of the micropores, as seen in PdAu/MP-SAPO-5 (Table 1). Further, it is has previously been shown that the presence of larger mesopores, in hierarchical systems, aids mass-transport to the catalyst surface and pore-diffusion to and from the active site [38,39]. In doing so, this can improve catalytic activity compared to purely microporous species, which may also be a secondary factor. It is possible that subtle differences in NP characteristics between the PdAu/HP-SAPO-5 and PdAu/MP-SAPO-5 could contribute to the differences in activity. However, given the similarity in NP size, shape, and metal environment (XPS and XAS) between these two systems, this is unlikely.

Figure 5b compares the catalytic response of bimetallic PdAu/HP-SAPO-5 with the monometallic Au/HP-SAPO-5 and Pd/HP-SAPO-5 catalysts. The monometallic Au/HP-SAPO-5 and Pd/HP-SAPO-5 catalysts showed inferior product yields of 1.2% and 0.4% benzaldehyde, respectively. The monometallic Au/HP-SAPO-5 catalyst displayed enlarged NPs with a particle size of 12.8 nm (Figure S7) and yielded inferior catalytic yields of 1.2%, compared to the bimetallic analogue. As the textural characteristics of the hierarchical catalysts are similar (Table 1), we believe the smaller, optimal particle sizes and bimetallic nature lead to synergistic enhancements in catalytic activity. Figure S8 compares the structure-property relationship of monometallic and bimetallic catalysts supported on hierarchical HP-SAPO-5 and microporous MP-SAPO-5. Despite the particle sizes of the bimetallic catalysts on the microporous and hierarchical supports being similar, a particle size of 8.5 nm for the bimetallic PdAu/HP-SAPO-5 catalyst produced superior reaction yields, compared to bimetallic PdAu supported on MP-SAPO-5, clearly illustrating the advantages of the hierarchical support. Preliminary results indicate that the PdAu/MP-SAPO-5 results in drastically reduced catalytic performances, on recycling, suggesting that NP aggregation and/or metal leaching during catalysis, could be a determining factor, but further work is required to substantiate this.

More importantly, to prove the efficacy and superior performance of the bimetallic Pd-Au catalyst, we performed a comparative physical mixture test, where the same molar equivalents of the monometallic Au/HP-SAPO-5 and Pd/HP-SAPO-5 were used in the catalytic reaction. It was noteworthy that this physical mixture of the two monometallic catalysts did not result in an increase in the catalytic performance but afforded a catalytic profile that is in line with their monometallic profile. On the other hand, the bimetallic PdAu catalyst anchored on HP-SAPO-5 afforded a 15-fold increase in catalytic performance, which vindicates the objectives of this study, confirming the proximity of the metals, or their dual inclusion in the synthesis process, plays a key role.

The in-depth understanding of the catalytic mechanism of alcohol oxidation over bimetallic nanocatalysts remains challenging to date. The oxidation of benzyl alcohol proceeds through a first oxidation step to benzaldehyde and subsequently through a second oxidation step to benzoic acid. The first step is crucial while the second step takes place very rapidly in polar solvents. It is important to carefully tune the selectivity of targeted product by adjusting the reaction conditions such as type of solvent, substrate concentration, nature of oxidant, type of metal and support interaction. The benzaldehyde formation pathway proceeds in three simple steps; first, the metal alkoxide formation by adsorption of alcohol on the metal NP. Second, β-hydride elimination takes place to form the carbonyl and hydride species. Finally, the metal hydride oxidised by the molecular oxygen to form a water molecule and regenerates the NP surface for adsorption and further catalytic cycles [56,57,58]. The Pd preferentially adsorbs the substrate molecule to form metal-alkoxide on the surface of NP [59]. This can be attributed to the higher electronegativity value of Au (χ = 2.54), which enhances the catalytic activity by decreasing the electron density of Pd (χ = 2.20). This electron deficient Pd is more susceptible to undergo oxidation to display superior catalytic performance than the monometallic catalysts. 

## 4. Conclusions

In summary, we have shown the controlled synthesis of a bimetallic PdAu catalyst, based on a hierarchical SAPO support, is effective for the catalytic oxidation of benzyl alcohol to benzaldehyde. The influence of dual porosity has been explored by comparing the performances of analogous hierarchical and microporous PdAu SAPO catalysts. The hierarchical PdAu/HP-SAPO-5 showed a 15-fold increase in catalytic activity compared to the analogous microporous PdAu/MP-SAPO-5. This is principally due to the hierarchical support retaining its porosity, on metal deposition, due to the inclusion of larger mesopores. The synergistic catalysis of PdAu NPs because of their electronic and geometric interactions has also been discussed by comparing it with a physical mixture variant of monometallic systems. The use of supported metal NPs in the oxidation reaction is sustainable and has economic and environmental benefits in contrast to the toxic oxidants. To the best of our knowledge, this work is the first example of immobilising PdAu NPs on HP-SAPO frameworks for sustainable oxidation reaction. Our future work will exploit the use of such unique hierarchical support materials, where we will tailor the composition and size distribution of bimetallic NPs to explore other reactions. Further optimization of synthetic protocols to contrast particle size distribution will facilitate more quantitative structure–activity relationships to be established. The comparative diffusion of substrate molecules in the HP and MP supports is ongoing. We hope that the investigation of distinctive support materials can pave the way for the design and synthesis of nanocatalysts in a variety of different heterogeneous catalytic application reactions.

## Figures and Tables

**Table 1:** Textural properties of the prepared catalysts determined by N_2_ physisorption analysis.

Catalysts	BET Surface Area (m^2^g^−1^)	Total Pore Volume (cm^3^g^−1^)	Microporous Pore Volume (cm^3^g^−1^)	Mesoporous Pore Volume (cm^3^g^−1^)	BJH Mesopore Diameter (Å)
HP-SAPO-5	299	0.24	0.06	0.18	53
Au/HP-SAPO-5	199	0.18	0.04	0.14	56
Pd/HP-SAPO-5	195	0.18	0.04	0.14	55
PdAu/HP-SAPO-5	194	0.17	0.04	0.13	56
MP-SAPO-5	271	0.14	0.12	0.02	--
PdAu/MP-SAPO-5	36	0.03	0.02	0.01	--

**Table 2:** ICP-MS analysis for the prepared catalysts.

Catalysts	ICP Experimental Metal Loading (Weight%)				
	Au	Pd	Al	P	Si
HP-SAPO-5	--	--	19.5	18.1	7.5
Au/HP-SAPO-5	0.6	--	19.6	19.9	9.6
Pd/HP-SAPO-5	--	0.4	19.3	18.0	7.6
PdAu/HP-SAPO-5	0.7	0.1	19.2	17.3	8.7
PdAu/MP-SAPO-5	0.6	0.2	19.6	19.7	3.6

## Data Availability

Not applicable.

## Supplementary Materials

The following are available online at https://www.mdpi.com/2079-4991/11/2/350/s1, Table S1: literature reports, Figure S1: N_2_ physisorption isotherms, Figure S2: pore size distribution, Figure S3: TEM micrograph of PdAu/HP-SAPO-5 along with the elemental mapping displaying the presence of Al, P, Si, Au and Pd, Figure S4: EDX spectrum of PdAu/HP-SAPO-5 displaying the presence of Al, P, Si, Au and Pd, Figure S5: UV-vis spectra of PdAu bimetallic catalysts on hierarchically porous and microporous SAPO-5, Figure S6: The (a) Au LIII-edge and (b) Pd K-edge XANES spectra of prepared catalysts Figure S7: TEM image and size distribution of Au/HP-SAPO-5, Figure S8: particle size and activity relationship, and Table S2: XPS B.E. shifts.
