# Supplementary material for: Bimetallic PdAu Catalysts within Hierarchically Porous Architectures for Aerobic Oxidation of Benzyl Alcohol
Source: Nanomaterials (Basel). 2021 Feb 1;11(2):350. doi: 10.3390/nano11020350 (PMC7912745; doi:10.3390/nano11020350)
Supplement: Supplementary file 1 [file nanomaterials-11-00350-s001.pdf]

nanomaterials

Supporting Information

Bimetallic PdAu Catalysts within Hierarchically Porous Architectures for Aerobic Oxidation of Benzyl Alcohol

Priyanka Verma, Matthew E. Potter, Alice E. Oakley, Panashe M. Mhembere and Robert Raja *

School of Chemistry, University of Southampton, University Road, Highfield, Southampton SO17 1BJ, UK; P.Verma@soton.ac.uk (P.V.); M.E.Potter@soton.ac.uk (M.E.P.); A.E.Oakley@soton.ac.uk (A.E.O.); P.M.Mhembere@soton.ac.uk (P.M.)

* Correspondence: r.raja@soton.ac.uk Tel.: +44-2380-599-542

Table S1. Benzyl alcohol oxidation to benzaldehyde using various catalysts.

Catalyst	Type of metal	Type of support	NPs size	Reaction conditions	Catalytic activity (%)		Reference
					Conv	selectivity	
Au/Fe_2O_3	Au	Fe_2O_3	--	Toluene, 2 bar O_2, 100 °C	7.1	87.6	[1]
Au/TiO_2	Au	TiO_2	--	Toluene, 2 bar O_2, 100 °C	0.65	100	[1]
Au/TiO_2-NR	Au	TiO_2 nanorod	6.2	K_2CO_3, Toluene, O_2, 100 °C	30	900	[2]
Au/SiO_2	Au	SiO_2	--	Toluene, 2 bar O_2, 100 °C	2.4	94.3	[1]
Au/C	Au	Carbon	--	Toluene, 2 bar O_2, 100 °C	2.3	90.4	[1]
Au/CeO_2	Au	CeO_2	--	Toluene, 2 bar O_2, 100 °C	3.4	100	[1]
Au/CeO_2 NR	Au	CeO_2 nanorod	3.6	Toluene, O_2, 100 °C	89	94	[3]
Au/RGO	Au	Graphene oxide	5.4	H_2O, $NaHCO_3$, O_2, 100 °C	65	93	[4]
Ru/C	Ru	Carbon	--	Toluene, O_2, 50 °C	100	98	[5]
Pt/C	Pt	Carbon	3.5	Toluene, pO_2 = 150 psi, 120 °C	2.8	90.7	[6]
Pt/Carbon Hybrid	Pt	Carbon hybrid	2.83	KOH, Toluene, O_2, 80 °C	99	99	[7]
PdPt/C	Pd, Pt	Carbon	2.2	Toluene, pO_2 = 150 psi, 120 °C	14.8	84.7	[6]
AuPd-PVP	Pd, Au	--	2.7	O_2, 100 °C	14.8	90.5	[8]
PdAu/MSNs	Pd, Au	Silica	3.6	Toluene, O_2 0.5 MPa, 90 °C	98	98	[9]

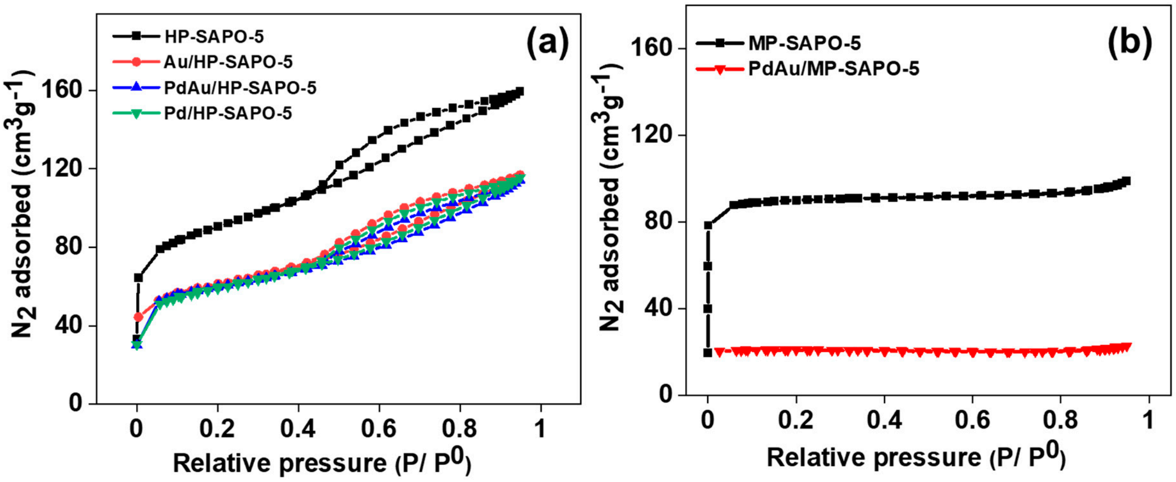

Figure S1. N_2 physisorption isotherms of prepared catalysts before and after NP deposition on (**a**) HP-SAPO-5 and (**b**) MP-SAPO-5.

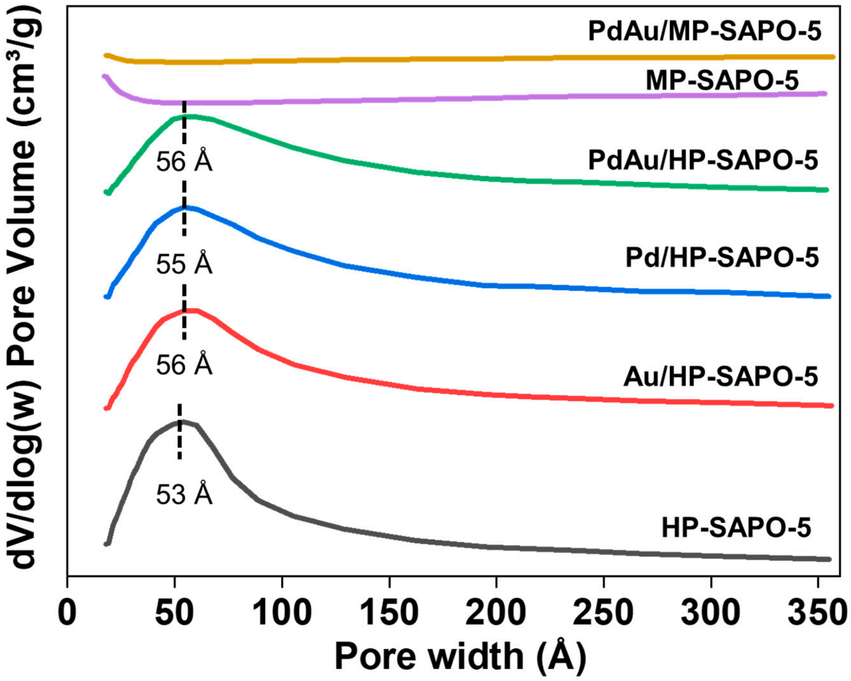

Figure S2. BJH pore size distribution for the hierarchically porous (HP) and microporous (MP) support systems before and after NP deposition. Plots are stacked for clarity.

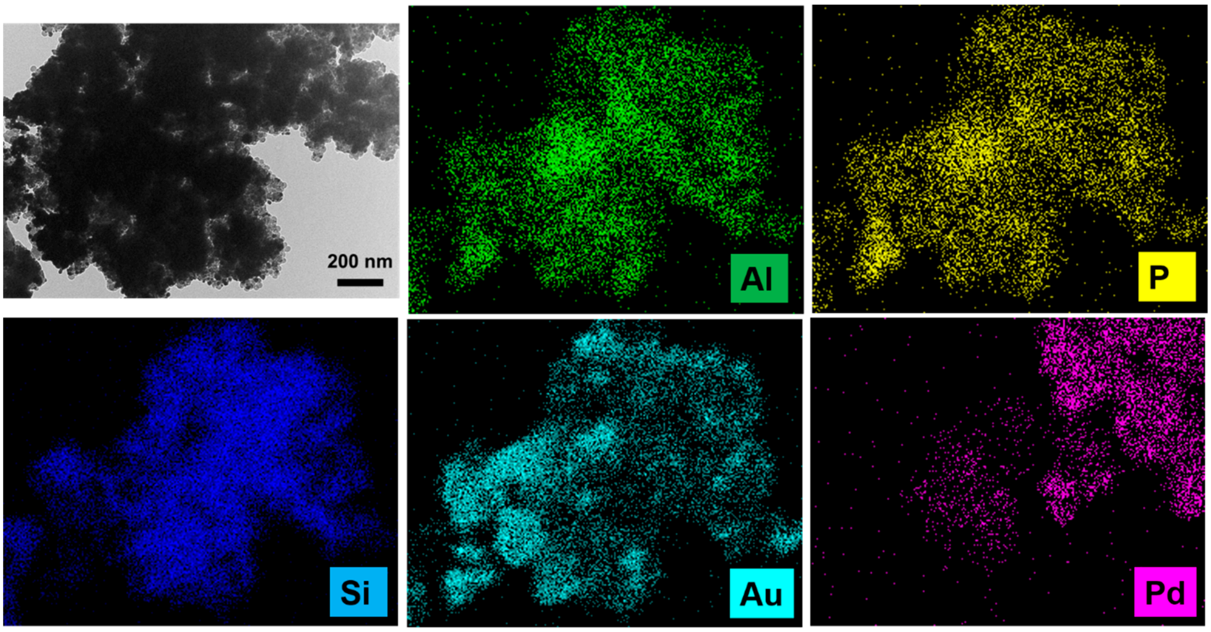

Figure S3. TEM micrograph of PdAu/HP-SAPO-5 along with the elemental mapping displaying the presence of Al, P, Si, Au and Pd.

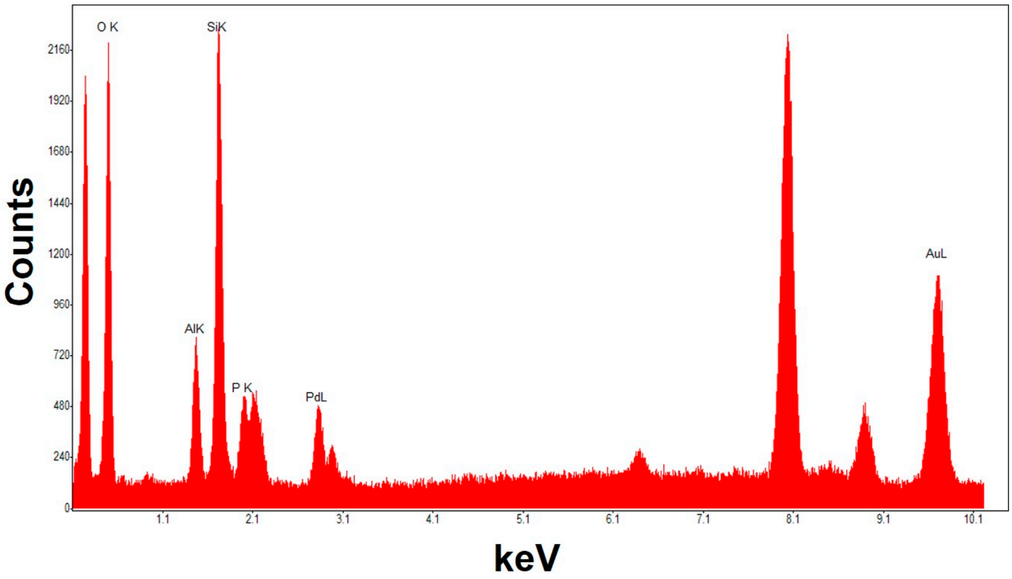

Figure S4. EDX spectrum of PdAu/HP-SAPO-5 displaying the presence of Al, P, Si, Au and Pd.

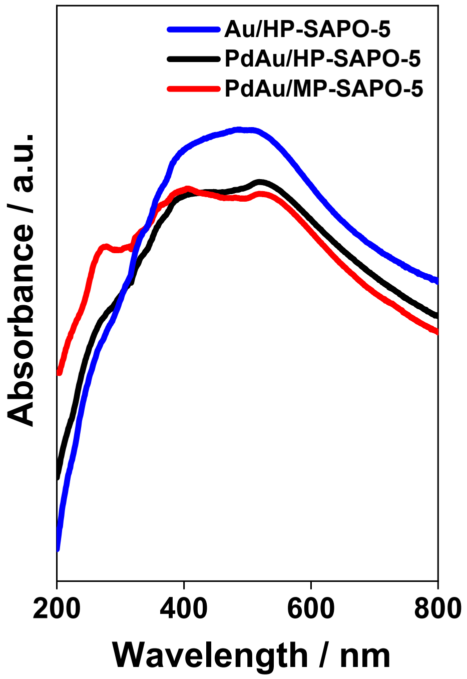

Figure S5. UV-vis spectra of PdAu bimetallic NPs on hierarchically porous and microporous SAPO-5.

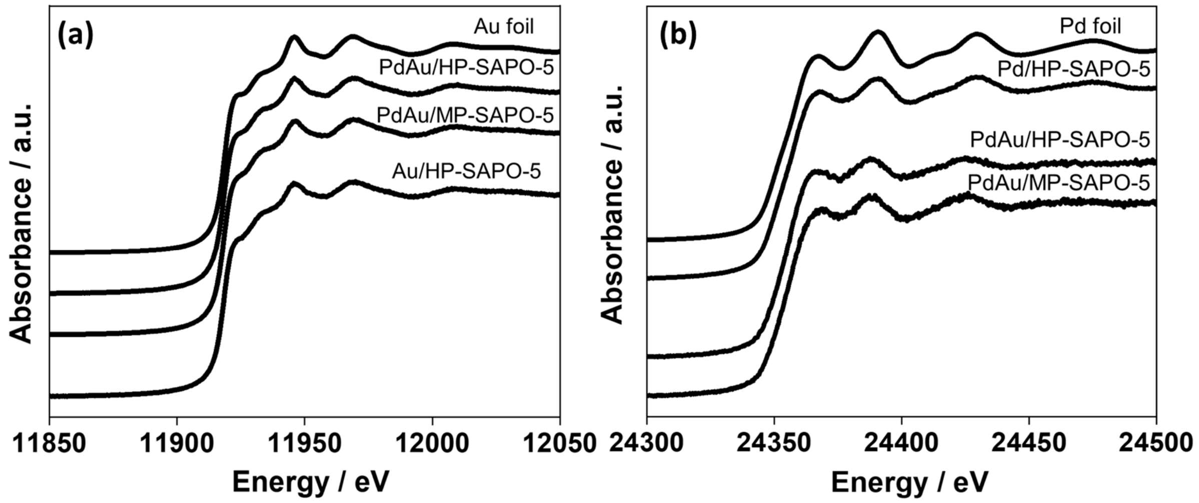

Figure S6. The (**a**) Au L_{III}-edge and (**b**) Pd K-edge X-ray absorption near edge structure (XANES) spectra of prepared catalysts.

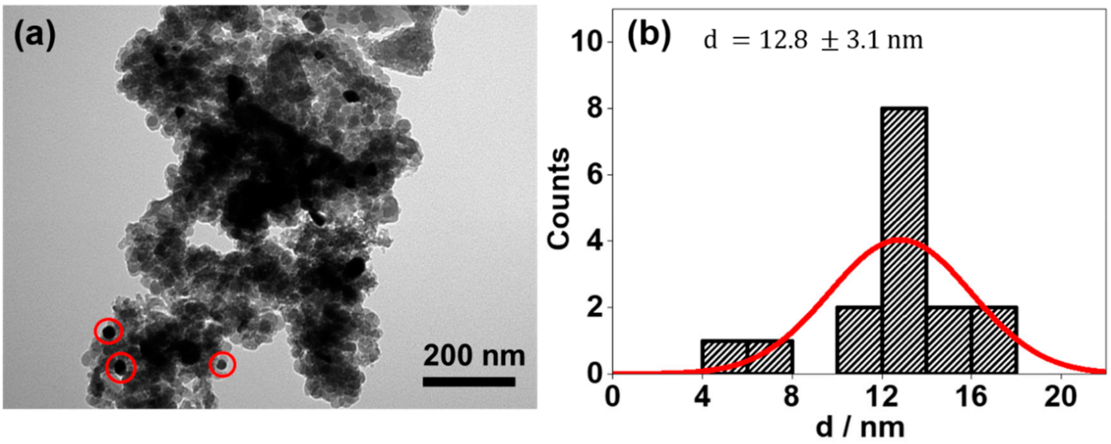

Figure S7. (**a**) TEM image and (**b**) size distribution of Au/HP-SAPO-5.

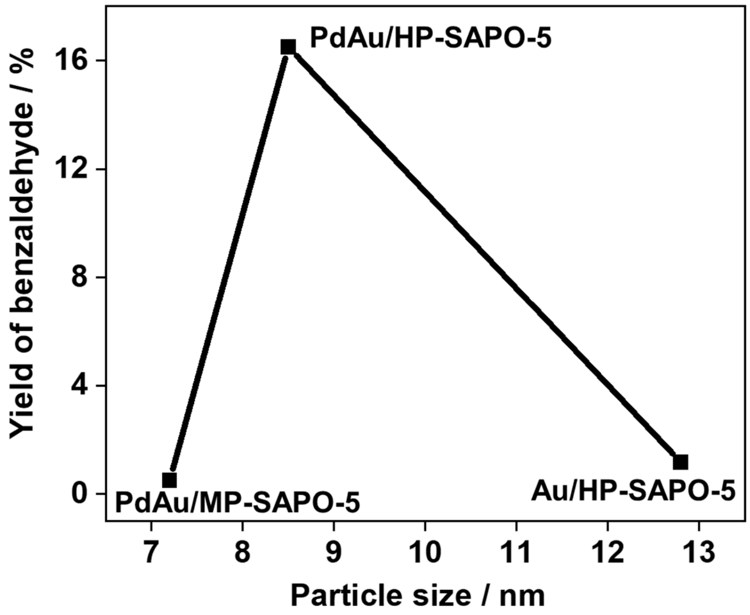

Figure S8. Relationship between particle size and catalytic yield for monometallic and bimetallic catalysts.

Table S2. The obtained binding energy (B.E.) values in the Au 4f and Pd 3d XPS spectral analysis.

Sample	Au 4f B.E. (eV)		Pd 3d B.E. (eV)	
	$4f_{7/2}$	$4f_{5/2}$	$3d_{5/2}$	$3d_{3/2}$
Au/HP-SAPO-5	84.0	87.6	--	--
Pd/HP-SAPO-5	--	--	335.2	340.4
PdAu/HP-SAPO-5	83.6	87.3	335.4	340.6
PdAu/MP-SAPO-5	83.8	87.5	335.5	340.7

References:

1. Enache, D.I.; Knight, D.W.; Hutchings, G.J. Solvent-free oxidation of primary alcohols to aldehydes using supported gold catalysts. *Catal. Letters* **2005**, *103*, 43–52.
2. Verma, P.; Mori, K.; Kuwahara, Y.; Cho, S.J.; Yamashita, H. Synthesis of plasmonic gold nanoparticles supported on morphology-controlled TiO2 for aerobic alcohol oxidation. *Catal. Today* **2020**, *352*, 255–261.
3. Lei, L.; Liu, H.; Wu, Z.; Qin, Z.; Wang, G.; Ma, J.; Luo, L.; Fan, W.; Wang, J. Aerobic Oxidation of Alcohols over Isolated Single Au Atoms Supported on CeO2 Nanorods: Catalysis of Interfacial [O-Ov-Ce-O-Au] Sites. *ACS Appl. Nano Mater.* **2019**, *2*, 5214–5223.
4. Yu, X.; Huo, Y.; Yang, J.; Chang, S.; Ma, Y.; Huang, W. Reduced graphene oxide supported Au nanoparticles as an efficient catalyst for aerobic oxidation of benzyl alcohol. *Appl. Surf. Sci.* **2013**, *280*, 450–455.
5. Dimitratos, N.; Lopez-Sanchez, J.A.; Hutchings, G.J. Selective liquid phase oxidation with supported metal nanoparticles. *Chem. Sci.* **2012**, *3*, 20–44.
6. He, Q.; Miedziak, P.J.; Kesavan, L.; Dimitratos, N.; Sankar, M.; Lopez-Sanchez, J.A.; Forde, M.M.; Edwards, J.K.; Knight, D.W.; Taylor, S.H.; et al. Switching-off toluene formation in the solvent-free oxidation of benzyl alcohol using supported trimetallic Au-Pd-Pt nanoparticles. *Faraday Discuss.* **2013**, *162*, 365–378.
7. Göksu, H.; Burhan, H.; Mustafov, S.D.; Şen, F. Oxidation of Benzyl Alcohol Compounds in the Presence of Carbon Hybrid Supported Platinum Nanoparticles (Pt@CHs) in Oxygen Atmosphere. *Sci. Rep.* **2020**, *10*, 1–8.
8. Hou, W.; Dehm, N.A.; Scott, R.W.J. Alcohol oxidations in aqueous solutions using Au, Pd, and bimetallic AuPd nanoparticle catalysts. *J. Catal.* **2008**, *253*, 22–27.
9. Yang, X.; Huang, C.; Fu, Z.; Song, H.; Liao, S.; Su, Y.; Du, L.; Li, X. An effective Pd-promoted gold catalyst supported on mesoporous silica particles for the oxidation of benzyl alcohol. *Appl. Catal. B Environ.* **2013**, *140–141*, 419–425.
